# Pavement Crack Detection from Mobile Laser Scanning Point Clouds Using a Time Grid

**DOI:** 10.3390/s20154198

**Published:** 2020-07-28

**Authors:** Mianqing Zhong, Lichun Sui, Zhihua Wang, Dongming Hu

**Affiliations:** 1 College of Geological Engineering and Geomatics, Chang’an University, Xi’an 710054, China; 2016026011@chd.edu.cn (M.Z.); 2017126029@chd.edu.cn (D.H.); 2 State Key Laboratory of Resources and Environment Information System, Institute of Geographic Sciences and Natural Resources Research, CAS, Beijing 100101, China; zhwang@lreis.ac.cn

**Keywords:** mobile laser scanning, pavement cracks, crack shape parameters

## Abstract

This paper presents a novel algorithm for detecting pavement cracks from mobile laser scanning (MLS) data. The algorithm losslessly transforms MLS data into a regular grid structure to adopt the proven image-based methods of crack extraction. To address the problem of lacking topology, this study assigns a two-dimensional index for each laser point depending on its scanning angle or acquisition time. Next, crack candidates are identified by integrating the differential intensity and height changes from their neighbors. Then, morphology filtering, a thinning algorithm, and the Freeman codes serve for the extraction of the edge and skeleton of the crack curves. Further than the other studies, this work quantitatively evaluates crack shape parameters: crack direction, width, length, and area, from the extracted crack points. The *F*1 scores of the quantity of the transverse, longitudinal, and oblique cracks correctly extracted from the test data reached 96.55%, 87.09%, and 81.48%, respectively. In addition, the average accuracy of the crack width and length exceeded 0.812 and 0.897. Experimental results demonstrate that the proposed approach is robust for detecting pavement cracks in a complex road surface status. The proposed method is also promising in serving the extraction of other on-road objects.

## 1. Introduction

Pavement cracking serves as a vital quantitative reference index for road maintenance [1,2]. Currently, large-scale efforts of manual inspection of cracks are rare owing to their low efficiency, high cost, and exposure to vehicle traffic [3,4]. Automatic detection and quantification of pavement cracks from road surface images and videos acquired using a variety of special sensors is becoming more common [5,6,7]. Various methods of crack recognition have been developed based on image processing and computer vision [8]. Excellent performances have been achieved in recent years since the successful application of neural networks and deep learning technology for image segmentation [9,10,11,12,13,14,15,16,17,18,19]. However, road surface images and videos are often obscured by light, shadows, stains, rust, and noise, making crack recognition challenging. Moreover, two-dimensional (2D) detection technology is limited regarding the illustration of the terrain and details of road damage, which reduces the performance of crack evaluation. Researchers have been exploring the methods for three-dimensional (3D) pavement crack detection and quantification. For example, the application of a line–structured light scanner and 3D camera can collect the detailed 3D terrain textures (with an accuracy of 1 mm or better) of pavement profiles [20,21,22,23,24,25,26]. However, the advanced 3D measurement systems that integrate numerous technologies are expensive, which hinders their large-scale application in pavement inspection [27].

In recent years, mobile laser scanning (MLS) systems have been gradually applied to fast, high-precision road and pavement condition surveys, and some researchers have investigated the applicability of MLS point clouds to crack extraction. Two kinds of algorithms were summarized in Reference [28]: (1) Projecting dense 3D point clouds into 2D images and extracting cracks using image-based algorithms [29], and (2) identifying cracks directly from a discrete point cloud. For example, Guan et al. [30,31] interpolated the point cloud into geo-referenced feature (GRF) images with ground resolution of 2 cm by applying a modified inverse distance-weighted (IDW) algorithm. Then, they picked out five test areas that contained a variety of cracks, ranging in width from a few centimeters up to 10 cm. The low-intensity pixels were selected as possible cracks using the maximum entropy threshold algorithm and enhanced by tensor voting to highlight the crack curves. The authors compared their results with that from pavement images. Their method was verified as effective for pavement cracks with low contrast and bad continuity and demonstrated much better performance compared to road surface images and videos. However, its computational time is considerable and mostly consumed by the iterative tensor voting process. Moreover, points with low intensities may be due to wet pavements, low-reflectance repair materials, stains, or other noises. In their study, Chen and Li rasterized all the ground points into a 4 cm digital terrain model (DTM) by selecting the minimum height within the grid as the pixel value [32]. Next, they used a high-pass filter to convolute the DTM to detect local elevation change. Finally, pavement cracks were delineated using a two-step matched filter. This method was computationally efficient and simple when compared to Guan’s. Convoluting a 2500 × 760 DTM only took a few seconds. Another advantage of the proposed method was that it successfully detected some cracks of only a few millimeters width. However, slight cracks are difficult to distinguish from the normal pavement height. In addition, it is difficult to select the grid size to retain the features, such as the crack width, needed for crack quantification. Without dimension reduction, Yu et al. [33] extracted 3D crack skeletons directly from mobile LiDAR point clouds. They normalized the intensities of the point data and identified crack candidate points with lower intensities using Otsu’s thresholding method. Next, they grouped crack-lines using a Euclidean distance clustering method. Finally, they extracted 3D crack skeletons by applying a L1-medial skeleton extraction method. This method executed quickly because it avoided time-consuming neighborhood searches in dense and discrete point clouds. However, crack points only account for a very small proportion of MLS data, causing Otsu’s threshold to be unreliable regarding segment cracks.

From all the research above, cracks often exhibit low-intensity value and can be detected by the intensity-threshold segmentation method. What is more, the method of intensity-threshold segmentation is independent of neighborhood contexts. Therefore, it can be applied to both images and point clouds. However, distinguishing cracks using an intensity threshold is not always reliable when encountering certain interference factors, such as uneven intensity, road markings, stains, low-reflectance mending materials, and sensor noise. The methods that interpolate dense 3D point clouds into 2D images can provide the neighbors information to delineate cracks by elevation changes. However, some cracks are too shallow to be detected by MLS data with a coordinate resolution of the mm-level. The completeness and robustness of extracted cracks only by low-intensity values or elevation changes may be insufficient. Also, high-resolution rasterization of massive point clouds consumes time and resources. More importantly, using a fixed size grid to interpolate point cloud weakens the quantitative evaluation performance of crack width.

This study attempts to propose a novel algorithm to detect 3D pavement cracks directly from MLS data and to quantitatively evaluate their shape parameters. To adapt to a noisy background, the method selects crack candidates based on the integration of the intensity difference and height changes from their neighbors. To address the problem of lacking topology in MLS data, this paper introduced a time grid (Tgrid) for the data collected by the 2D scanner with the same incremental angles. Each point is assigned a row and column number based on either its scan angle or acquisition time. Thus, through a point’s row and column numbers, it is easy to find its neighbor points. Moreover, with the regular grid indexes, MLS data can be processed like images, and then crack detection can be performed using proven image-based methods. From this, the Tgrid can also serve the detection of other on-road objects from MLS data, such as curbs, road markings, and manholes. Moreover, it offers promising potential for application in convolutional neural networks and deep learning with MLS point clouds. The proposed method was tested in road conditions over a 220 m stretch of road with hundreds of cracks. The remainder of this study is organized as follows: Section 2 introduces the materials and details the proposed method; Section 3 presents the results and discussion; and Section 4 presents our conclusions.

## 2. Materials and Methods

### 2.1. Materials

**Figure 1:**
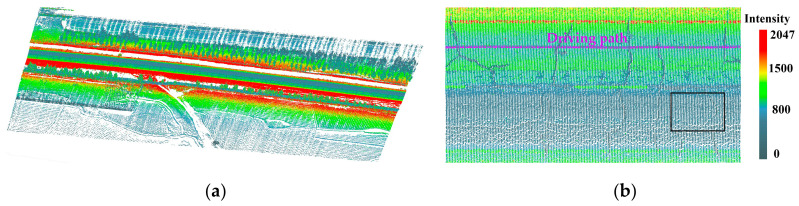
The test data. (**a**) The overall view of test data, and (**b**) the divergent point intensity in two lanes.

The test dataset shown in Figure 1a was collected using the SSW-IV vehicle laser modeling and measurement system, including an approximately 220 m long highway with numerous cracks. A 2D scanner with a 360° field of view (FOV) was assembled at the rear of an SUV roof 2.2 m above the road surface and oriented perpendicular to the moving direction. The 2D scanning plane was set at an angle of 45 degrees inclined from the horizontal plane. The scanner provided 500,000 measurements and 200 scan lines per second, featuring a detection range of up to 300 m. The concrete road surface was measured at a speed of 30 km/h, resulting in the spacing between adjacent scan lines of approximately 4 cm and distance between adjacent laser points of 0.7–3 cm in the road. Figure 1b shows the common phenomenon of divergent point intensity in two lanes. The point intensities of the lane on the other side were so low (black box shown in Figure 1b) that some cracks were difficult to distinguish with a fixed intensity threshold. In addition, the road surface contained road markings, ruts, stains, black spots, and other intensity-altering factors, which would have an unfavorable effect on algorithms that are based on a global intensity threshold. This situation indicates that the use of point intensity differences and altitude changes to the background may be more effective than a fixed intensity threshold alone.

### 2.2. Methods

**Figure 2:**
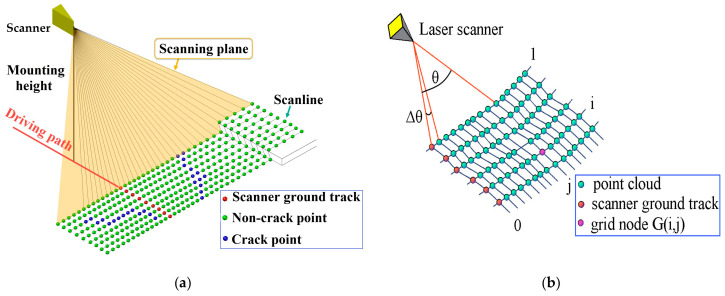
The connectivity provided by the arrangement of mobile laser scanning (MLS) point clouds. (**a**) The arrangement of MLS point clouds collected by a two-dimensional (2D) scanner with equal incremental angle. (**b**) Create a Time grid (Tgrid) for MLS point cloud by using the scanning line and scan angle information.

As a linear feature, cracks should be continuous; however, discrete MLS points have no connectivity. This study attempts to identify crack points from their neighbors, which are defined by an especially topological structure within which the discrete MLS points are grid-adjacent. The introduced structure is built based on the imaging mechanism of the MLS system. Figure 2a sketches the arrangement of the MLS point cloud collected over time using a 2D scanner with equal incremental angles. The laser beams are projected onto different scan lines as the vehicle moves. The neighbors of the crack points on the same scan line can be simply identified according to the collection sequence of the point data, whereas neighbors across multiple scanning lines can be deduced using similar scanning angles. Based on the above assumptions, we created a 2D grid index for MLS point clouds, as shown in Figure 2b. We named the structure Time grid (Tgrid) because it records the arrangement of the MLS point cloud over time.

The proposed method comprises three steps: (i) Construction of the Tgrid for the MLS point clouds, (ii) detection of road surface point and crack candidates, and (iii) generation of the crack skeleton and calculation of the crack shape parameters.

### 2.3. Construction of a Tgrid for MLS Point Clouds

Let P(*X*, *Y*, *Z*, *T*, *I*) represent the test datasets with N points, where (*X*, *Y*, *Z*) denotes the spatial coordinates of a point, and *T* and *I* express its acquisition time and intensity. The Tgrid records the 2D arrangement of MLS points along and across the scan lines. Each point is assigned a grid index (*i*, *j*), from which it can search for its neighbors. A point with the index (*i*, *j*) is located on the *i*th scanning line, and its scanning angle θ is *j* times the angular resolution ∆θ. The values of *j* can be positive or negative.

**Figure 3:**
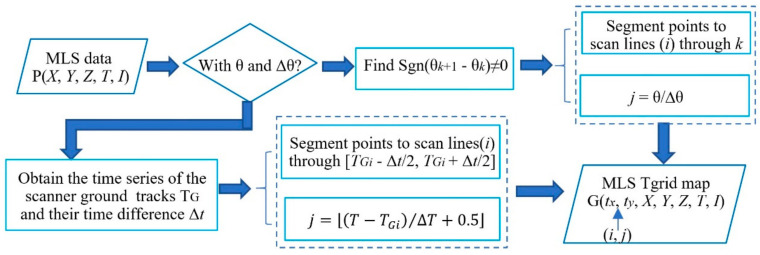
The flow chart of construction of a Tgrid for MLS data.

This study provides two approaches to construct a Tgrid using scanning angle (θ) or acquisition time (*T*). Figure 3 shows a flow chart of the two approaches.

**Figure 4:**
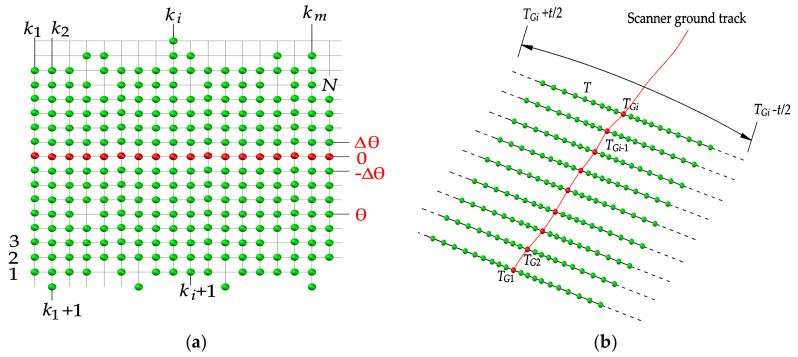
Construction of a Tgrid for MLS data through (**a**) the scan angle (θ), and (**b**) the acquisition time (*T*).

As shown in Figure 4a, the scan angle, θ, increases or decreases monotonously with *T* along the same scan line. Thus, we divided the point clouds to their scanning lines by detecting the sign change of the angle difference between the adjacent laser points. When the neighboring point satisfies Sgn(θ*_k_*_+1_ − θ*_k_*) ≠ 0, *k* = 1, 2, …, N, the *k*th point is a piecewise point. Let *m* represent the number of *k* detected. The numerical array [1, *k*_1_, *k*_2_, …, *k_m_*, N] then forms (*m* + 1) non-overlapping intervals, which correspond to (*m* + 1) scan lines. On each scanning line, the multiples of scan angles, θ, with respect to the angular resolution, ∆θ, is calculated to obtain the *j* value of each point, *j* = θ/∆θ.

When θ is not available or the MLS data provides a scan angle level with insufficient accuracy, the above method is ineffective. In such a situation, we propose a different approach of constructing Tgrid based on *T*. In our previous study [34], we deduced that the time series of the scanner ground track, i.e., the laser points that the scanner projects directly below, should be in accordance with an isochromatic sequence. As shown in Figure 4b, let T_G_ be the time series of the scanner ground track, T_G_ = {*T_G_*_1_, *T_G_*_2_, *T_G_*_3_, …, *T_Gm_*_+1_}, and *T_Gi_*_+1_ − *T_Gi_* = Δ*t*, where Δ*t* is the elapsed time on each scan line. The time range [*T_Gi_* − Δ*t*/2, *T_Gi_* + Δ*t*/2] divides the laser points to the *i*th scanning line. The *j* indexes of points on *i*th scanning line can be derived as $j=\lfloor \left(T-T_{Gi}\right)/\Delta T+0.5\rfloor$, where Δ*T* denotes the emission time interval of the laser beams. If ∆*T* is unknown, it can be estimated using the time difference (*T_i_*_+1_ − *T_i_*) between the adjacent laser points. Note that (*T_i_*_+1_ − *T_i_*) may change over time. We use the symbol ⌊  ⌋ to reduce the estimation error (for more detail, please refer to Reference [34]). In the Tgrid, each point (*i*, *j*) has a maximum of four side neighbors: (*I −* 1, *j*), (*i* + 1, *j*), (*i*, *j −* 1), and (*i*, *j* + 1), and four diagonal neighbors: (*i −* 1, *j −* 1), (*i* + 1, *j −* 1), (*i −* 1, *j* + 1), and (*i +* 1, *j* + 1). The spatial position of these neighbors is free. In addition, eight neighbors may not always exist. When there are no laser beam returns and no neighbors, they are shown as empty nodes (as shown in (*i −* 1, *j −* 1) in Figure 2b).

For easy understanding, we added two columns (*x_t_*, *y_t_*) before P(*X*, *Y*, *Z*, *T*, *I*) to the expressed MLS point clouds as a Tgrid map, G(*x_t_*, *y_t_*, *X*, *Y*, *Z*, *T*, *I*), where (*x_t_*, *y_t_*) represents the row and column of a point in the Tgrid map. Similar to a multi-band image, (*X*, *Y*, *Z*, *T*, *I*) represent the multi-band characteristics. The cost of building and maintaining such a 2D grid is quite low.

### 2.4. Detection of Road Surface Points and Crack Candidates

#### 2.4.1. Detection of Road Surface Points

**Figure 5:**
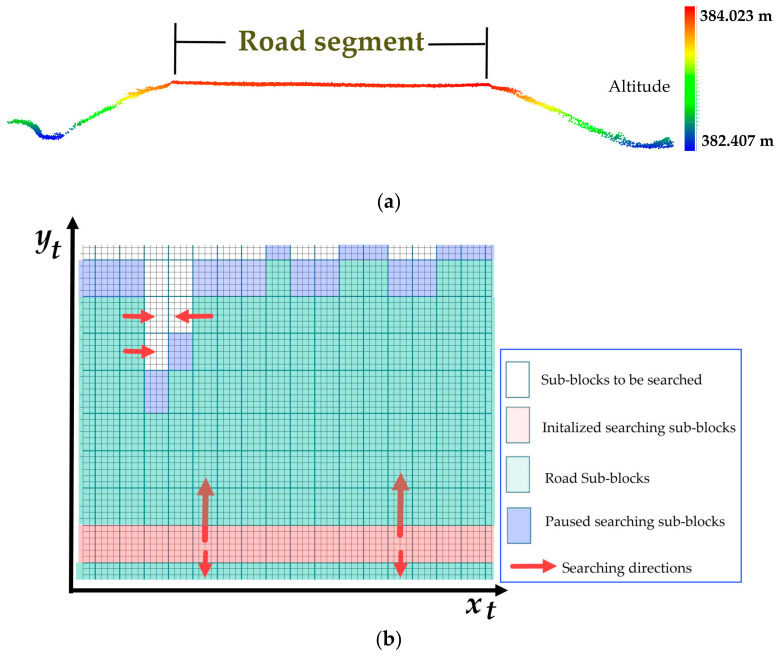
Detection of road surface and boundary area in Tgrid. (**a**) The cross-section of a road segment, and (**b**) the searching process of a road boundary area.

**Figure 6:**
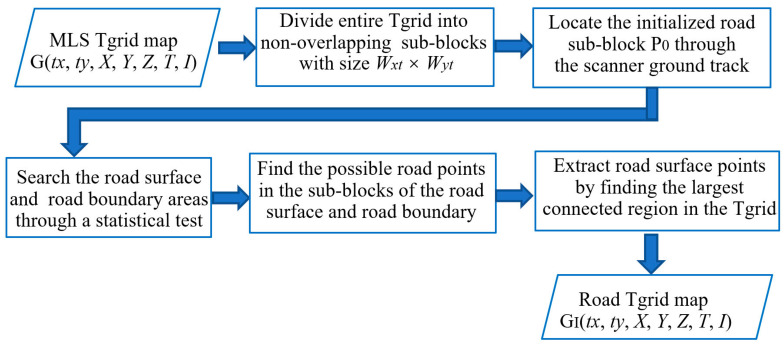
The flow chart of the method of detection of road surface points.

Various algorithms have been developed to extract roads from MLS point clouds. The advantage of using the Tgrid is that the road boundaries can be defined as a continuous curve. Figure 5a shows the cross-section of the studied road with natural slopes on both sides. To identify road surface points quickly, we used the method in Reference [35], which roughly approximated the local road patch as a 3D best-fitting plane. Next, this method took the trajectory data as the initial road seed to search the road boundary area along and perpendicular to the scan line using a statistical hypothesis testing method based on the point altitudes. Figure 6 shows a flow chart of the method of detection of road surface points. First, the entire Tgrid is divided into non-overlapping sub-blocks, the size of which is W*x_t_* × W*y_t_*. The sub-block located by the scanner ground track serves as the initialized searching sub-block, P_0_ (shown in red in Figure 5b). Next, both the road surface and road boundary areas are searched along *x_t_* and *y_t_* directions through a statistical hypothesis test based on the point altitudes in the sub-block. Then, the possible road points are extracted from the sub-blocks of the road surface and road boundary and finally, identified by finding the largest connected region in the Tgrid.

Assume the point altitude, *Z_S_*, in a road patch conforms to the Gaussian distribution of N (μ, σ) and that μ is unknown. Let the number of points within the sub-block be *N_b_*. A statistical test problem for the population variance with unknown mean is as follows:

(1) $$H_{0}:\;\sigma^{2}\;\geq \;\sigma_{0}{}^{2},\;H_{1}:\sigma^{2}\;<\;\sigma_{0}{}^{2}$$

(2) $$\bar{Z}=\frac{1}{N_{b}}\overset{N_{b}}{\underset{i=1}{\sum }}Zs_{i}$$

(3) $$s^{2}=\frac{1}{N_{b}-1}\sum_{i=1}^{N_{b}}{\left(Zs_{i}-\bar{Z}\right)}^{2}$$

where $\bar{Z}$ denotes the mean value and *s*^2^ is the standard deviation. The statistical variable and its region of rejection are:

(4) $$\chi^{2}=\frac{\left(N_{b}-1\right)}{\sigma_{0}{}^{2}}s^{2}$$

(5) $$\chi^{2}\leq \chi^{2}{}_{\left(1-\alpha \right)}$$

where α is the significance level or the confidence; generally, α = 0.05. If the statistical variable of the sub-block is located inside of the region of rejection, H_1_ is accepted (shown in green in Figure 4b) and H_0_ is denied. This means that no road boundary area is found; otherwise, H_0_ is accepted (shown in blue in Figure 4b). The associated sub-block is selected for the road boundary area. Figure 4b illustrates the process of detecting this area. The proposed method can be summarized as follows:

(1) Set an initialized searching sub-block, P_0_, where trajectory data are located, and calculate the statistical variance, σ_0_^2^, of the point altitudes within it.
(2) Search forward to find the road boundary area along the direction of *x_t_*, *y_t_*, and the opposite *x_t_* and opposite *y_t_,* until a sub-block (shown in blue) that passes the test is found. Pause searching.
(3) Iterate for all trajectory points until all road boundary areas are beside each other.
(4) Filter the non-road points in the road boundary area (shown in blue) and its closed road surface neighbors (the closed sub-block shown in green) using a height threshold, *h*_th_, to the local road plane.
(5) Extract the complete road surface by finding the largest connected region in the Tgrid.

The size of the sub-block (W*x_t_* × W*y_t_*) can be determined through the geometric dimension, *W_D_*. Although the distance of the adjacent points is inconsistent in *y_t_* directions, we unified the *W_yt_* for convenience. The mean spacing between the adjacent scanner ground track, $d_{s}$ (in *x_t_* direction), and the largest distance between the adjacent road grid points, $d_{a}$ (in *y_t_* direction), are used to determine W*x_t_* and W*y_t_*. This means that *Wx_t_* =$\left\lfloor \;W_{D}/d_{s}+0.5\right\rfloor$ and *Wy_t_* = $\left\lfloor W_{D}/d_{a}+0.5\right\rfloor$. Due to the fixed size of the sub-blocks, it is also possible that the road boundary lies in the road sub-block adjacent to the boundary area. Thus, we derive the local road plane by fitting the 3D coordinates of the points in a road boundary area (shown in blue) and its closed road neighbors (the closed sub-block shown in green) using the robust least-squares method. Hence, more road points are participating in the fitting of the local plane. The conformity of the fitting plane to the road surface is thereby ensured. The height of points involved in the fitting operation to the local plane is taken as the evaluation standard of whether this is a non-road point. The height threshold, *h*_th_, can be given according to the roughness of the road surface.

#### 2.4.2. Detection of Crack Candidates

The crack points are detected by two approaches: detection (1) of the valley point that is lower than local pavement and (2) of the low-intensity point with height no higher than the road surface. 

(1) Detection of the valley point

**Figure 7:**
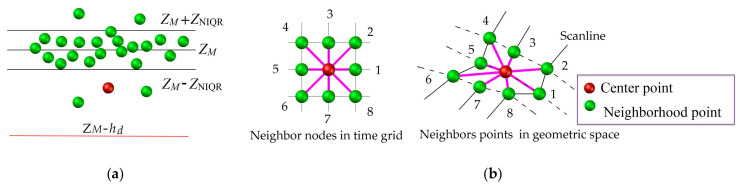
Characteristics of valley points. (**a**) Height feature, and (**b**) gradient effect near crack candidates.

The valley point has two typical characteristics: a height difference exceeding the road surface roughness and a nearby gradient change. We defined a circular neighbor region with radius, *L_R_*, that is larger than the common maximum width of the crack. An M estimator of Andrew’s type was applied to derive the robust altitude, *Z_M_*, of the road surface and the standard interquartile interval, *Z*_NIQR_, is used to measure the degree of the pavement roughness. The two conditions are combined as follows:

(6) $$\forall G\left(i,j\right):\left\{\begin{array}{c} \begin{array}{cc} if(Z\left(i,j\right)>Z_{M}–h_{d}) & penetration \end{array}\;point\\ \begin{array}{cc} if(Z\left(i,j\right)>Z_{M}–h_{d}\;\&\;Z\left(i,j\right)\langle Z_{M}-Z_{\mathrm{NIQR}}\;\&\;\mathrm{grad}f\left(i,j\right)\rangle v_{th}) & valley\;point \end{array}\\ \begin{array}{cc} otherwise, & irrelevant\;point \end{array} \end{array}\right.$$

where *h_d_* expresses the maximum possible depth of the crack, which is designed to eliminate the penetration laser point caused by loose pavement facilities, such as rainwater grates, well covers, etc. Next, grad*f*(*i*, *j*) represents the average gradient value centered on (*i*, *j*), which is calculated through the geometric coordinates (*X*, *Y*, *Z*) of eight neighbors using Equation (7). Note that only the existing neighbors are calculated. Following this, *v_th_* defines the minimum gradient change requirement of the crack candidates. Figure 7 depicts the characteristics of the valley points. The extracted valley points were recorded to dataset G_V_:

(7) $$\mathrm{grad}f(i,\;j)=\left|\frac{1}{k}\sum_{1}^{k}\left(\mathrm{gradient}\left(i,j,k\right)\right)\right|\;\mathrm{gradient}\left(i,j,k\right)\;=\frac{Z\left(i,j,k\right)-Z\left(i,j\right)}{\sqrt{{\left(X\left(i,j,k\right)-X\left(i,j\right)\right)}^{2}+{\left(Y\left(i,j,k\right)-Y\left(i,j\right)\right)}^{2}}}$$

where *k* is the number of the existing neighbors centered on (*i*, *j*).

(2) Detection of low-intensity points with height no higher than the road surface

Some slight cracks have only a few points with the valley effect, and further candidates are points with low intensities. However, the intensity of the road surface points varies greatly owing to many factors. The low-intensity point clouds do not necessarily belong to cracks, and vice versa. In addition, the proportion of the crack points is so small that it is difficult to segment through a threshold automatically provided by the commonly used method. Our approach is to first detect the low-intensity points and then filter out those that are higher than the road surface, thereby retaining the points with low-intensity that are no higher than the road surface.

**Figure 8:**
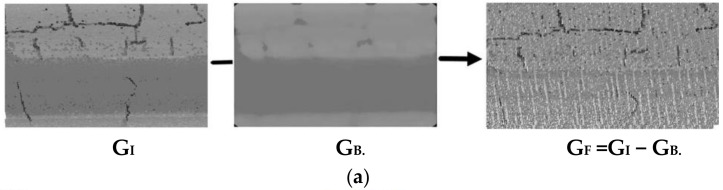

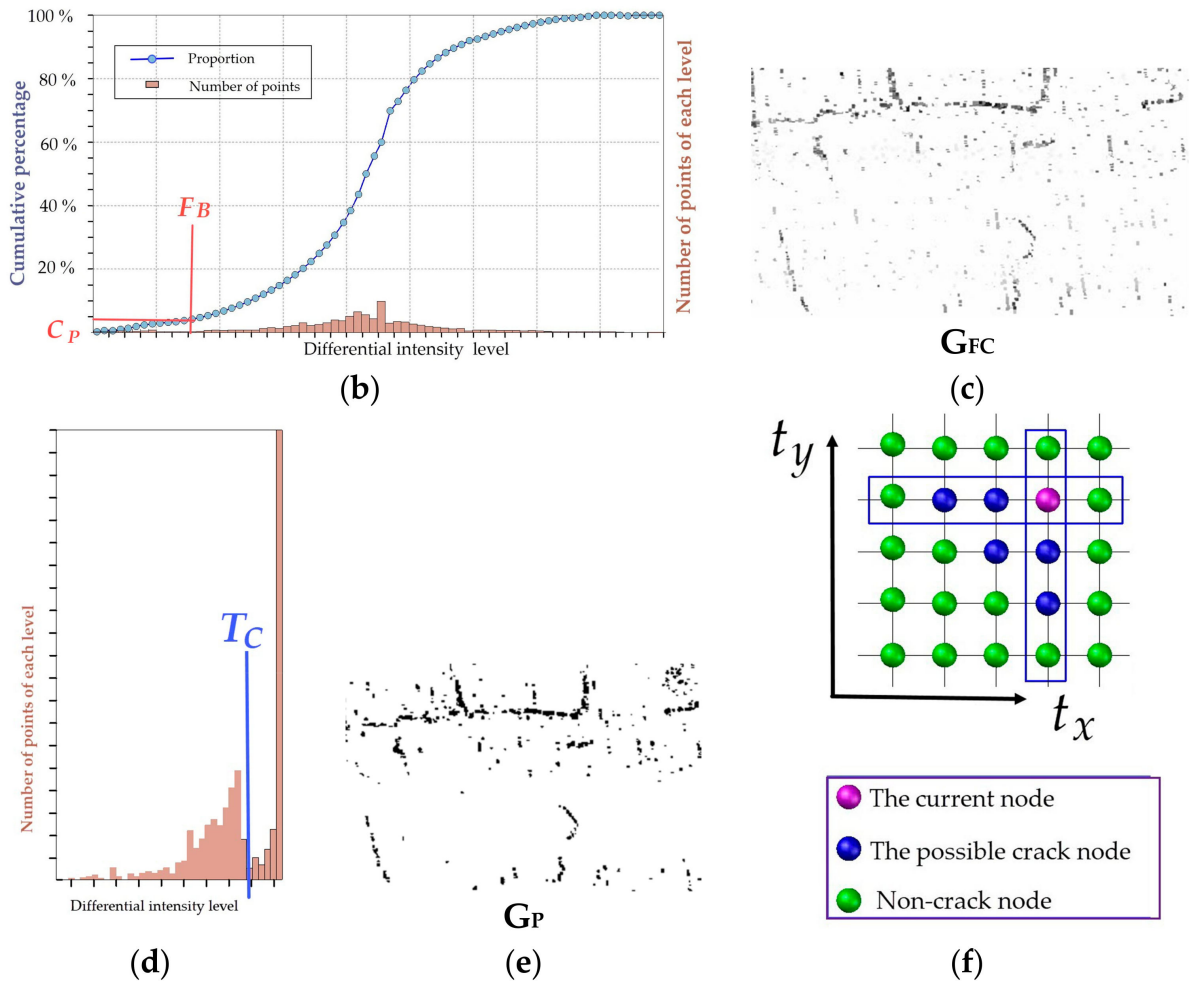
The flow of detection of the low-intensity points with height no higher than the road surface. (**a**) The background-differential map (G_F_), (**b**) the relevant truncation value (*F_B_*) by the given proportion of cracks’ point (*T_P_*), (**c**) the differential truncation map (G_FC_), (**d**) the segment threshold, *T_C_*, of the possible cracks, (**e**) the binary map of possible crack candidates (G_P_), and (**f**) the filter about valley effect.

This study detects the lower intensity points through a background-differential map, within which the crack proportion limitation (*T_P_*) is preset to reduce the unfavorable effects of any generous non-crack points. Figure 8 shows the flow of the detection method.

We first generated the background map, G_B_, using a median filter from the input intensity Tgrid map, G_I_, which derives from the grid index (*i*, *j*) and its intensity. Next, the background-differential map, G_F_, is obtained using a minus operation as follows: G_F_ = G_I_ − G_B_. In Figure 8b, the proportion limitation of the crack points (*C_P_*) is given and the relevant truncation value, *F_B_,* is calculated on the histogram of G_F_. The differential truncation map, G_FC_, is generated in Figure 8c by transforming the value of G_F_(*i*,*j*) that is higher than *F_B_* to *F_B_*. The maximum entropy method is then adopted to determine the segment threshold, *T_C_*, of the possible crack points (recorded in G_P_) and non-crack points. Finally, G_P_ is filtered through the valley effect to remove the nearest non-crack point on both sides of the G_P_ point along the *t_x_* and *t_y_* directions to compare their altitude. As shown in Figure 8f, two rectangles frame the crack points in the directions of *t_x_* and *t_y_* respectively, with non-crack points intercepted at two ends when centered on a candidate. If the altitude of the candidate is no greater than the largest of the four non-crack neighbors, the candidate is reserved; otherwise, it is filtered out. The remaining crack candidates are saved to the binary map, G_C_.

Some crack candidates obtained by these two approaches may overlap. Thus, the union of G_V_ ∪ G_C_ is taken as the candidate crack points.

### 2.5. Generation of the Crack Skeleton and Calculation of Crack-Shape Parameters

#### 2.5.1. Generation of the Crack Skeleton

The extracted crack candidates may be noisy and discontinuous. Thus, a series of morphological operations are implemented to extract the continuous crack skeleton. First, to eliminate small holes and connect the nearest candidates, the morphology closing algorithm is adopted to cluster the crack regions. Next, the one-point-thick continuous crack curves are extracted using the thinning algorithm proposed in Reference [36]. Following this, the burrs and branches are separated according to the connectivity of the points on the skeleton.

**Figure 9:**
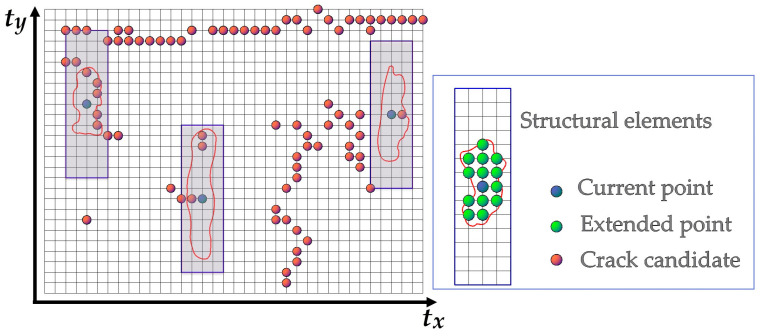
The structural elements of the morphological closing algorithm.

Morphological image processing techniques are often used to connect crack fragments into crack curves and for complicated crack shapes in image-based methods [2,37]. Unlike the geometric grid, the Euclidean distance of the adjacent points on the Tgrid is constantly changing. The size of structural elements of the closing algorithm is not fixed but is defined by a geometric dimension. As shown in Figure 9, we set a double-layer search structure, the first layer of which is a rectangle with a fixed size, and the second layer of which is a preset geometric search circle with the radius *D*_2_. Given that the width and the height of the rectangle are *R_W_* and *R_H_*, they can be determined by the distance between the densest point cloud under the vehicle in the direction of *x_t_* and *y_t_.* We assumed these to be *d_x_* and *d_y_*, respectively. Thus, *R_W_* = 2 × $\left\lfloor D_{2}/d_{x}+0.5\right\rfloor$ + 1, *R_H_* = 2 × $\left\lfloor D_{2}/d_{y}+0.5\right\rfloor$ + 1. The points within the second layer form the structural elements of the morphology algorithm. Note that some empty nodes without geometric coordinates may be expanded during this process. To mark the connected regions of the cracks using the 8-connected neighborhood, the 3D coordinates of the expanded empty nodes are linearly interpolated using the closest existing neighbors at both sides along the *t_y_* direction.

To remove the isolated noise, we extract the maximum width (represented by the number of points in the *t_x_* direction) and height (the number of points in the *t_y_* direction) of the connected regions and recover them to the geometrical space to calculate their geometrical dimensions. If the longest of two sides is larger than the set length threshold, *L_th_*_1_, the connected region indicates a curve region. Otherwise, it is marked as noise to be removed. The remaining regions are thinned to extract the continuous crack skeletons. 

To remove the burrs, the connectivity of points in the skeletons are distinguished as endpoints, general skeleton points, and intersections, which correspond to points that are single-connected, dual-connected, and connected by no less than three. The burrs are recognized by the space distance threshold, *L_th_*_1_, between the endpoint and the intersections that were first encountered. Each skeleton only leaves one longest main curve, and the rest are defined as branches, i.e., another skeleton. The skeleton curve is traced by the Freeman chain code and the coordinates of crack points are recorded to a table to calculate the crack-shape parameters.

#### 2.5.2. Calculation of Crack-Shape Parameters

The crack-shape parameters here include crack direction, width, length, and area.

(1) The crack direction

**Figure 10:**
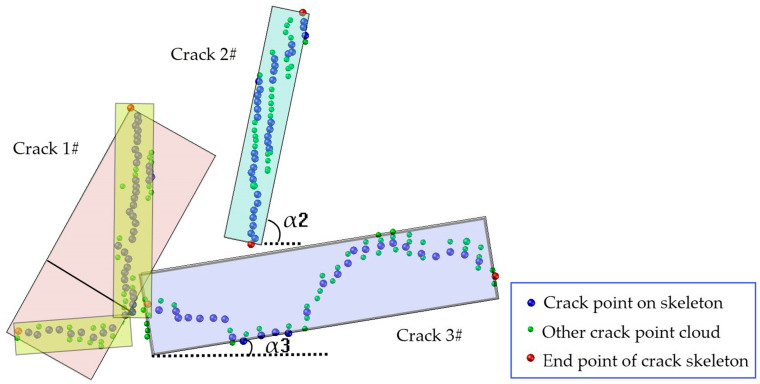
The judgment of the crack direction.

To determine the crack direction, the crack points and skeleton are recovered to the geometric space. We use the length/width ratio *R_LW_* of the smallest circumscribed rectangle of the skeleton points and its long axis direction to determine the crack direction. When *R_LW_* exceeds the set threshold, *R_LWth_*, the direction of the crack must be evaluated in sections. Otherwise, the crack is oriented by the angle α between the long axis direction of the minimum circumscribed rectangle and the vehicle path. The crack direction is classified as transverse, vertical, or oblique with the related inclination ranges of the α value [0, π/6], (π/6, π/3), and [π/3, π/2]. In Figure 10, crack 2# and crack 3# are classified as transverse and vertical due to the qualified *R_LW_* and the scope of the values of α. Crack 1# has an *R_LW_* exceeding the specified threshold and is further separated into two sections, the piecewise points of which are given at the farthest point from the straight-line connecting head and tail. The *R_LW_* ratio of the smallest external rectangle of the cracks is recalculated and split again until *R_LW_* ≤ *R_LWth_*, and the angle α between the long axis direction and the path line is taken to identify the trends of the segmented cracks.

(2) The crack width

The minimum distance method is often used to calculate the crack width in image-based approaches; that is, to take the shortest distance from a point on one edge to the other as the crack width at this edge point. Equation (8) expresses the calculation, where *w_i_* corresponds to the width of the *i*th point of the left edge. Next, *j* = 1, 2, 3, …, N_R_, where N_R_ denotes the number of points on the right edge. However, limited by the geometric resolution of the MLS data, the extracted cracks contain many single-point-thickness points, particularly the transverse cracks along the scan line:

(8) $$w=\min\;(\sqrt{{\left(X_{i}-X_{j}\right)}^{2}+{\left(Y_{i}-Y_{j}\right)}^{2}})$$

**Figure 11:**
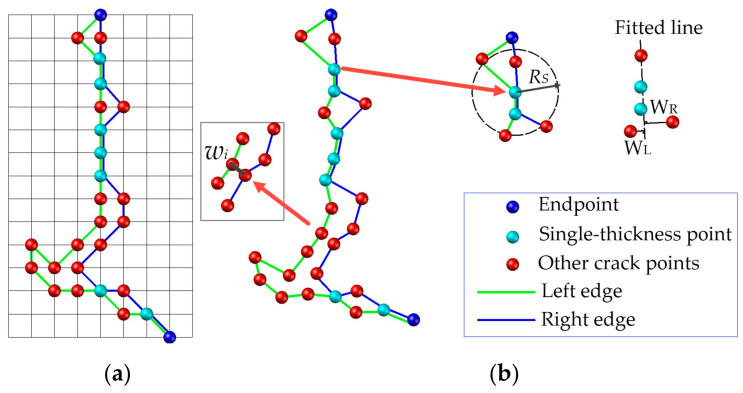
Edge points classification to calculate crack width, (**a**) in Tgrid, and (**b**) in geometrical space.

This study selects the single-point-thickness edge point, from which a search radius, *R_S_*, is given to fit the edge points within the search range to a virtual edge line. The maximum distances *w_iL_* and *w_iR_* from the edge points to the line on both sides are calculated, and *w_i_* = max (*w_iL_*, *w_iR_*) is taken as the related width of the point. The procedures of the crack width calculations are as follows:

- In the Tgrid, the Freeman chain code is used to track the edge of the crack connection area. The closed edge curve is disconnected from the endpoint of the skeleton to split the edge into the left and right borders (Figure 11a).
- The edge points shared by the left and right borders are searched and marked as single-point-thickness edge points. In the above method, *w_i_* = max (*w_iL_*, *w_iR_*) is adopted to calculate the related crack width (Figure 11b), while other edge points use Equation (8).
- Output the average width of all edge points to measure the severity of the cracks. The maximum width of the crack and its corresponding location, P_m_(*i*, *j*, *X*, *Y*, *Z*, *T*, *I*), serve as the supplementary information.

(3) The crack length 

The crack length is calculated by accumulating the distance of the adjacent points from the crack skeleton using Equation (9). Here, the complex network of cracks is evaluated separately from the main skeleton curves and branches:

(9) $$L_{D}=\sum_{k=1}^{N_{L}-1}\sqrt{{\left(X_{k+1}-X_{k}\right)}^{2}+{\left(Y_{k+1}-Y_{k}\right)}^{2}}$$

where (*X_k_*, *Y_k_*) and (*X_k_*_+1_, *Y_k_*_+1_) express the plane coordinates of two adjacent crack skeleton points, assuming there are *N_L_* skeleton points in the crack.

(4) The crack area 

The crack area is obtained by adding up the total area of the triangulated irregular network (TIN) produced by the crack edge points. For the single-point-thickness region, the TIN is flattened. The single-point-thickness region can be roughly estimated using the length of the skeleton multiplied by the average width of the corresponding point.

## 3. Results and Discussion

The pavement cracks in the test data were automatically extracted on a computer with 16 GB RAM and 4 CPU cores (each @3.6 GHz). Table 1 lists the optional parameters used in the process. The parameters *W_D_*, *α*, and *h_th_* in Section 2.4.1 represent the geometric dimension of the initialized searching sub-block P_0_, the significance level or the confidence of the statistical test problem, and the roughness of the road surface, respectively. The parameters *h_d_* and *v_th_* express the maximum possible depth and the minimum gradient change requirement of the valley point. The parameter *C_P_* in Section 2.4.2 presets the proportion of the crack points in the number of all point clouds. The parameter *D*_2_ is the preset searching radius of the second layer of the structural elements in the morphological closing algorithm. The parameter *L_th_*_1_ denotes the minimum length threshold of the connected region of crack points. The parameter *R_lwth_* in Section 2.5.2 is the threshold of the length/width ratio to determine the crack direction. The parameter *R_s_* illustrates a search radius to define a virtual edge line when dealing with the single-point-thickness edge point.

**Figure 12:**
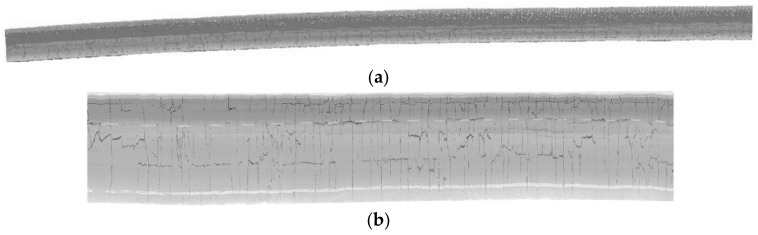
The extracted point cloud of the road surface and time grid map rendered with intensity. (**a**) The extracted point cloud of the road surface, and (**b**) the intensity-based Tgrid map of the road surface.

Figure 12 shows the intensity Tgrid map of the road surface, of which each point with the regular numbers of rows and columns illustrates the point adjacency. As shown, there are many transverse and longitudinal cracks in the pavement, and the width of the longitudinal cracks is expanded near the path but compressed far from the path due to the change of geometric resolution along the scanning line. Moreover, the transverse cracks in the Tgrid map are extremely narrow and often appear to be of single-pixel-thickness.

**Figure 13:**
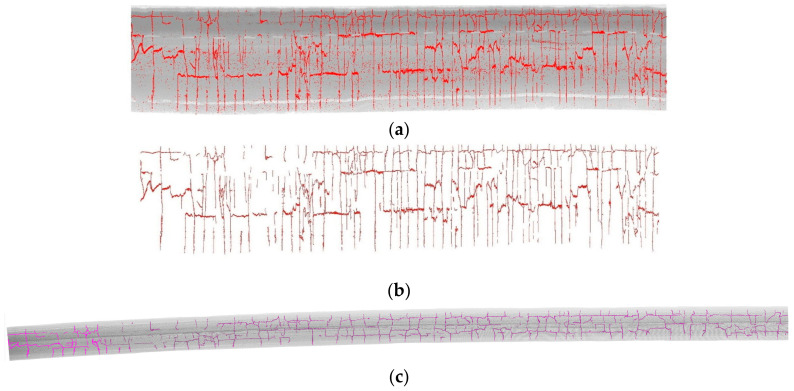
The extracted crack candidates in the Tgrid map and the results of crack craves. (**a**) The extracted crack candidates (in red) on the intensity-based Tgrid map of the road surface, (**b**) the remaining crack candidates after Morphological filtering, and (**c**) overlay of crack craves (in magenta) and road surface point cloud.

Figure 13a shows the extracted crack candidates, which contain significant noise. Figure 13b expresses the remaining crack points after morphological filtering and Figure 13c overlies the crack curves to the road surface. To enhance the display effect, the crack points are magnified in Figure 11.

For a quantitative assessment of the results, we obtained the ground truth by means of manual measurement and field investigation. Due to the geometric resolution of the MLS data, we compared only the extracted cracks where the width was above the minimum calculated value (approximately 1 cm). The number of crack curves detected, those not detected, and the error detection were counted to calculate the precision (*P*) and recall (*R*). Precision (*P*) represents the proportion of all “correctly detected items” to all “actually detected”. Recall (*R*) refers to the proportion of all “correctly detected items” to all “should be detected”. To evaluate the quality of the proposed method, we used a common evaluation standard, the *F*1-Measure (also known as *F*1 score), that combines the results of precision (*P*) and recall (*R*). The quotas of *P*, *R*, and *F*1 are calculated using Equations (10) and (11), respectively. Table 2 shows the quantitative results of the proposed method on these three types.

(10) P=TP(TP+FP)R=TP(TP+FN), 

(11) $$F1=2\frac{P\times R}{P+R},$$

where *T_P_* is the true positive, i.e., the correctly detected crack curves, while *F_P_* expresses the false positive, i.e., the number of cracks detected incorrectly. *F_N_* denotes false negative, i.e., the number of crack curves not detected.

As shown in Table 2, the performance of detection for the three types of cracks varies greatly. Taking the *F*1 score as the evaluation standard, the scores of transverse crack, longitudinal crack, and oblique crack are 96.55%, 87.09%, and 81.48%, respectively. Among them, the transverse crack has the highest detection rate (*R*), while the *R* is relatively poor for the longitudinal and oblique cracks (less than 80%). This means that the cracks distributed along the scanning line were detected successfully (*R* = 98.00%), and the cracks across multiple scanning lines went undetected. In addition, the false detection rate of transverse and longitudinal cracks was very low. This score was followed by that of the oblique cracks. This result may be related to the aggregation effect of crack point distribution on the Tgrid map, in which the points of transverse and longitudinal cracks were basically clustered vertically and horizontally while the points of oblique crack were distributed in a relatively scattered fashion. These distributions resulted in poor performance with the lowest detection rate and the highest error detection rate. Finally, three potholes were mistakenly identified as cracks.

**Figure 14:**
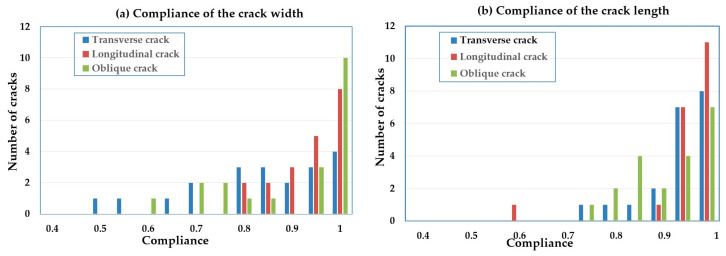
The histogram of length and width compliance of cracks in 60 samples. (**a**) Compliance of the crack width, and (**b**) compliance of the crack length.

To quantify the accuracy of the crack extraction, 60 curves were selected to test the width and length conformance of three types of cracks with 20 curves for each type. Equation (12) was used to calculate the width and length conformance, represented by *C_W_* and *C_L_*:

(12) $$\left\{\begin{array}{c} C_{w}=1-\left|T_{w}-E_{w}\right|/T_{w}\;\\ C_{L}=1-\left|T_{L}-E_{L}\right|/T_{L}\;\; \end{array}\right.$$

where *T_W_* and *E_W_* represent the average width of the actual crack and extracted crack respectively, and *T_L_* and *E_L_* are the total length of the actual crack and extracted crack. Figure 14 shows the histogram of the width and length compliance of 60 extracted cracks at 0.05 intervals, in piles of three types of cracks.

The width compliance of the transverse cracks was relatively scattered and that of the longitudinal cracks was the most concentrated. For the length compliance, the oblique cracks were dispersed and the other two were relatively centralized. Table 3 shows the quantitative results of the 60 samples after averaging the compliance. The variance of the width /length compliance illustrates the above trends. Specifically, an incomplete detection of a longitudinal crack, as an abnormal case, increased the variance of this type.

From the mean value of *C_W_* and *C_L_*, the estimated width of the longitudinal crack was the closest to the actual width, followed by that of the oblique crack. In addition, the width compliance of the transverse curves was the lowest at only 0.812. This may be attributable to the fact that most of the transverse cracks are single-point-width cracks. With only one laser point on the width direction of the crack curve, it is difficult to obtain high-precision calculation results. The width accuracy of the oblique cracks was second, which may owe to its scattered distribution across multiple scan lines, bringing challenges to its identification and width measurement. The longitudinal crack points gather in the *t_x_* direction with high geometric resolution. With multiple points in the crack width direction, the longitudinal crack achieved the highest accuracy of the width measurement, i.e., up to 91.05%. It is worth noting that spot checks were also made on the widest section and related position of the extracted cracks. However, no satisfactory results were obtained. Considering the positioning error rate of the MLS system, a position error within ±5 cm is regarded as an accurate detection. Nonetheless, the correct detection rate of the widest section was less than 60%. It may be inferred that the geometric resolution requiring improvement is still the bottleneck of the crack detection from the MLS data.

The length error mainly manifested in the lack of extracted length. Some of the transverse, longitudinal, and inclined cracks suffered a portion of the length loss at the endpoint. There were some cases of one longitudinal or oblique crack splitting into two pieces with one section undetected. We regarded such cases as correct detections with insufficient length. Finally, no large gaps were found in the length compliance of the three types: all were approximately 90%.

**Figure 15:**
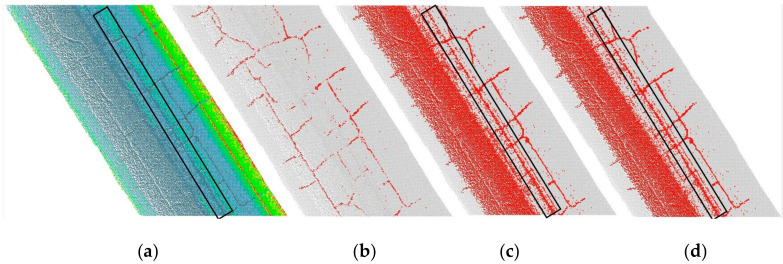
The detected crack candidates by using different methods. (**a**) Original point cloud, (**b**) the detected crack candidates by using the proposed method, (**c**) by using the maximum entropy sum method, and (**d**) by using Otsu’s thresholding method.

To further illustrate the merits of integrating the low-intensity and altitude changes for the detection of crack candidates, we conducted comparative tests. For comparison, we used Guan’s method that adopts the maximum entropy sum thresholding to detect possible crack pixels, and with Yu’s method that used Otsu thresholding. Unlike our proposed method, these other two methods only use intensity values. To have a unified basis for comparison, only the methods used to determine the threshold value could be compared. This excludes the use of the subsampling process to generate interpolation images. The results are shown in Figure 15.

It is evident that using Otsu’s threshold or the maximum entropy threshold extracted over-sized crack candidates. Although they are excellent methods for automatically determining the global threshold, they cannot adaptively adjust to the extraction of cracks with very small proportions in large-scale data ranges. Moreover, the proposed background difference algorithm improves the negative effects of inconsistent intensity due to road roughness, road markings, different pavement materials, etc. In addition, the ruts expressed as raised ridges are not distinguished by low intensities (the black box in Figure 15a,c,d). This study embeds the analysis of the altitude changes near the crack points: the ridged ruts have been removed and the robustness of the method has improved. When compared with the aforementioned threshold-segmentation methods, the proposed method is advantageous in its adaption to more complex environments and global applicability; thus, it effectively segments cracks from large-scale MLS point clouds. 

**Figure 16:**
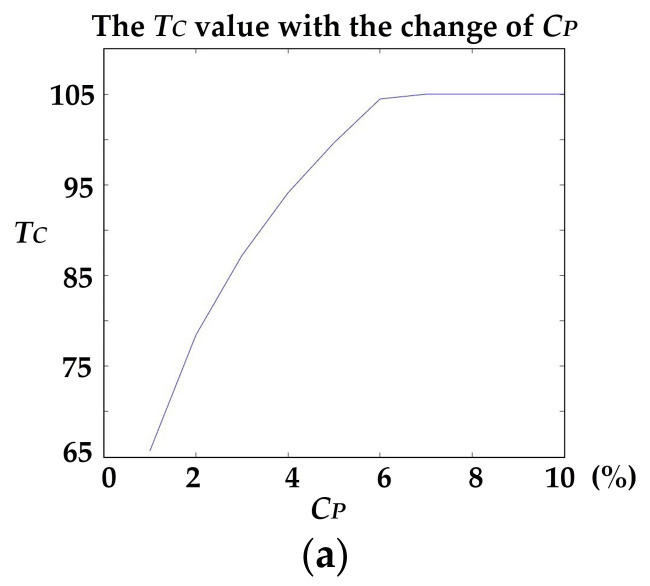

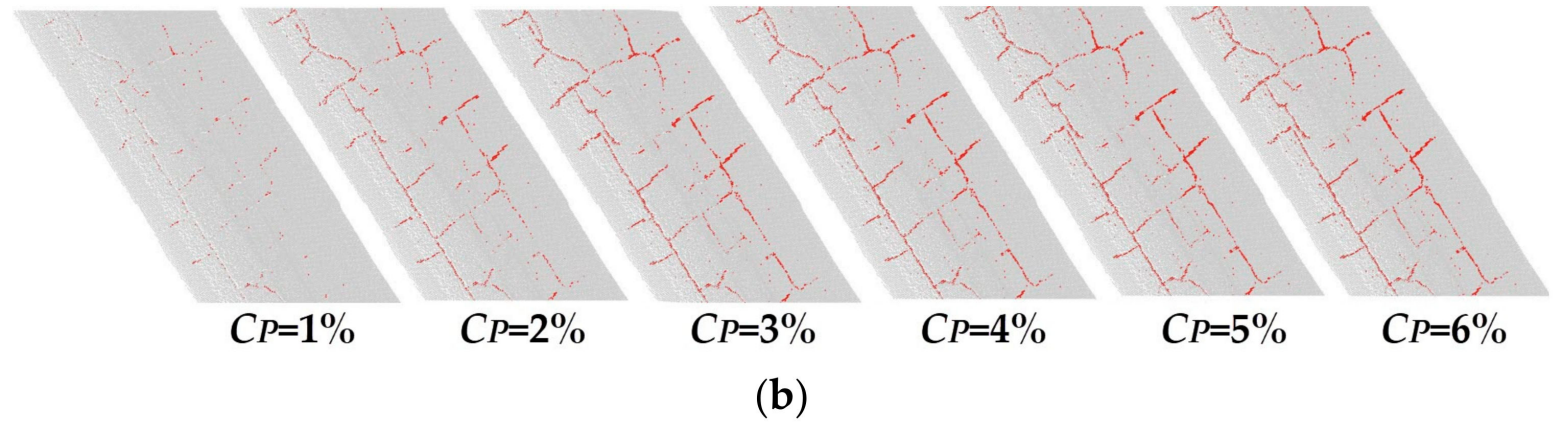
The sensitivity test of the *C_P_* with *T_C_*. (**a**) The change of the value of *T_C_* with *C_P_*, and (**b**) crack candidates corresponding to the selected *C_P_*.

Finally, the sensitivity of the *C_P_* with *T_C_* was also tested. Figure 16a shows the changing trends of the value of *T_C_* with *C_P_*; where *C_P_* increases, *T_C_* increases. When *C_P_* reaches 6%, *T_C_* approaches saturation. We did not test for cases greater than 10%, as crack points rarely account for more than 10% of the surface points on large roadways.

Although valuable (but not universal), interference-related noise caused by road markings, etc., that accounted for a larger proportion of the point cloud than cracks, is greatly reduced after setting *C_P_* (not exceeding 10%). It is also evident that when *C_P_* exceeds 4% (Figure 16b), the extracted crack curve is almost complete. As *C_P_* continues to increase, the noise increases. It is preferable to infer that the real proportion of cracks of the test data was approximately 4%. The results show that presetting the crack ratio is effective for eliminating interference.

The proposed method uses the regular grid indexes to transform discrete MLS point clouds into structures that are similar to multispectral images, from which the altitude and intensity information are integrated to detect the robust crack candidates. Image processing methods, such as morphological algorithms, can be used for post-processing. The execution time of building the Tgrid, detecting the valley points, and selecting points with low-intensity that are no higher than the road surface was 13.267 s, 8.9 s, and 78.21 s, respectively. The time cost of the proposed method is only one-thousandth that of Guan’s method of projecting 3D point clouds to 2D to construct geometric grid topology and using the tensor voting algorithm. Another benefit of the proposed method is that it does not lose the original accuracy of the point cloud and connects crack points using adjacency relation, both of which offer a sound basis for detecting cracks.

To summarize, the proposed method can successfully detect cracks with a width of not less than 1 cm using MLS data. As the geometric resolution of the state-of-the-art MLS system improves (the distance between adjacent points *t_x_* and *t_y_* direction can be compressed to 0.1–1 cm), detecting cracks from MLS data would have more potential and research value. In addition, the proposed structure makes point cloud processing easier and can introduce mature image processing methods into point cloud processing. More efforts are expected to be devoted to the detection of other on-road facilities, such as curbs, road markings, and manholes, from MLS data using the Tgrid method. Furthermore, the potential of Tgrid for improving the current deep learning point cloud frameworks is promising.

## 4. Conclusions

In previous studies, MLS data may not have been the first choice to evaluate pavement cracks owing to its geometric resolution and discrete distribution without connectivity. However, as the geometric resolution of the MLS system has been greatly improved, detecting cracks from MLS data has gained potential and significant research value. Based on the scanning angle or acquisition time of MLS point cloud, this study has losslessly transformed discrete MLS data into a “grid map” (Tgrid) through assigning each point cloud a grid index. Thus, numerous proven image-based methods of crack extraction can be implemented in point cloud processing. The approach was tested on a 220 m stretch of road with hundreds of cracks. This work has successfully constructed the “Tgrid map” for the test data and used the adjacency relation provided by this map to detect road surfaces, extract continuous road boundaries, and allow for image-based methods of identifying and quantifying pavement cracks in MLS point clouds. The results prove the feasibility and effectiveness of the proposed method on transverse, longitudinal, and oblique cracks, respectively. 3D crack skeletons and their shape parameters were extracted. The *F*1 scores of the quantity of correctly extracted transverse, longitudinal, and oblique cracks reached 96.55%, 87.09%, and 81.48%, respectively. Moreover, the average accuracies of the crack width and length were above 0.812 and 0.897. Experiments have proven that using the presented method, the MLS point cloud can be processed similarly to images. This finding is valuable for extraction of many on-road features, such as road points, curbs, road markings, and manholes, from MLS data. Furthermore, the proposed structure (Tgrid) can avoid the loss of information due to dimension reduction and has the potential to improve the existing deep learning framework for point clouds.

## Figures and Tables

**Table 1:** Parameters used in the process.

Parameters	Values	Parameters	Values	Parameters	Values
*W_D_* (m)	0.50	*v_th_* (°)	10	*R_lwth_*	3
*α*	0.05	*C_P_* (%)	5	*R_s_* (m)	0.20
*h_th_* (m)	0.03	*D*_2_ (m)	0.04		
*h_d_* (m)	0.20	*L_th_*_1_ (m)	0.25		

**Table 2:** Quantitative assessment of the proposed method on three types of cracks.

Crack Type	Actual Quantity	Precision(*P*)	Recall (*R*)	*F*1*-Measure*
Transverse crack	98	95.15%	98.00%	96.55%
Longitudinal crack	47	97.91%	78.43%	87.09%
Oblique crack	42	84.62%	78.57%	81.48%

**Table 3:** Quantitative evaluation of the width/length compliance of 3 types cracks.

Crack Type	Number of Samples	Mean (*C_W_*)	Variance (*V_W_*)	Mean (*C_L_*)	Variance (*V_L_*)
Transverse crack	20	0.812	0.149	0.921	0.066
Longitudinal crack	20	0.910	0.071	0.935	0.089
Oblique crack	20	0.874	0.131	0.897	0.076
